# Contribution of *Vouacapoua americana* fruit-fall to the release of biomass in a lowland Amazon forest

**DOI:** 10.1038/s41598-021-83803-y

**Published:** 2021-02-22

**Authors:** Victor Juan Ulises Rodriguez Chuma, Darren Norris

**Affiliations:** 1 Postgraduate Program in Tropical Biodiversity, School of Environmental Sciences, Federal University of Amapá, Rod. Juscelino Kubitscheck, km 02, Macapá, 68903-419 Brazil; 2 Ecology and Conservation of Amazonian Vertebrates Research Group, Federal University of Amapá, Rod. Juscelino Kubitscheck, km 02, Macapá, 68903-419 Brazil

**Keywords:** Biodiversity, Conservation biology

## Abstract

Fruit-fall provides the transfer of biomass and nutrients between forest strata and remains a poorly understood component of Amazon forest systems. Here we detail fruit-fall patterns including those of *Vouacapoua americana* a Critically Endangered timber species across 25 km^2^ of lowland Amazon forest in 2016. We use multi-model comparisons and an ensemble model to explain and interpolate fruit-fall data collected in 90 plots (totaling 4.42 ha). By comparing patterns in relation to observed and remotely sensed biomass estimates we establish the seasonal contribution of *V. americana* fruit-fall biomass. Overall fruit-fall biomass was 44.84 kg ha^−1^ month^−1^ from an average of 44.55 species per hectare, with *V. americana* dominating both the number and biomass of fallen fruits (43% and 64%, number and biomass respectively). Spatially explicit interpolations provided an estimate of 114 Mg dry biomass of *V. americana* fruit-fall across the 25 km^2^ area. This quantity represents the rapid transfer by a single species of between 0.01 and 0.02% of the overall above ground standing biomass in the area. These findings support calls for a more detailed understanding of the contribution of individual species to carbon and nutrient flows in tropical forest systems needed to evaluate the impacts of population declines predicted from short (< 65 year) logging cycles.

## Introduction

The study of the dynamics and distribution of forest fruiting is central for understanding forest regeneration and the dynamics of forest ecosystems^1–4^. This is a largely under-studied phenomenon, particularly in highly diverse Amazonian forests. Fruit-fall is the part of forest fruit production that is unused/missed by canopy frugivores^5^. The availability of fallen fruit therefore depends on not only reproductive phenology but also weather (wind and rain) and the plant-animal interactions with both canopy and terrestrial frugivores^1,5,6^. Fruit-fall biomass whilst not in absolute terms as important as timber provides an immediately available release of resources for myriad consumers that are key to maintaining the diversity of tropical forest systems^4,7,8^. Despite their importance, fruit-fall patterns are one of the myriad below canopy components of Amazon forest diversity that have yet to be adequately explained or predicted.

Although fruit production is a critical link for tropical forest biodiversity networks^3,4^ such ecological interactions are not included to inform logging quotas that establish the quantity and frequency of timber harvests or impact assessments that establish environmental compensation of logging activities across Amazon forests. Selective logging may reduce overall impacts on biodiversity, yet there is increasing evidence that the selective harvest of commercially attractive but rare emergent trees can degrade ecosystem services provided by tropical forests^9,10^. Large emergent trees (> 60 cm diameter at breast height) are typically rare but have a disproportionate effect on tropical forest diversity, carbon stocks and ecosystem services^3,11–13^. Additionally, ecologically ‘rare’ trees constitute majorities in commercially logged species assemblages and overly simplistic reduced impact logging quotas can lead to unsustainable declines in rare but commercially valuable species^14^.

Brazil retains 64% of the world’s total intact forest landscapes, of which 72% of natural forests are in Amazonia^15^. Yet growing political pressure is increasing the number and scale of legal forest logging concessions across the Brazilian Amazon including within protected areas^16,17^. Although managing yields of selectively-logged forests is crucial for the long-term integrity of forest biodiversity and financial viability of local industries, overly general quotas governing legal concessions can lead to the ecologic and/or economic extinction of target species^14,18,19^. A basin wide analysis of authorized logging in private and community-owned forests showed no evidence of recovery in total value of forest stands beyond the first-cut, suggesting that the commercially most valuable timber species become predictably rare or economically extinct^19^. Additionally due to climatic influences on tree mortality^20^ and growth^21^ droughts can substantially reduce stocks, biomass and timber recovery rates in areas managed under reduced impact logging regimes^22^.

The internationally valuable and Critically Endangered timber species *Vouacapoua americana*^23^ is unlikely to persist ecologically or economically under recommended reduced impact logging systems in Brazil. Although most valued for timber *V. americana* is an evolutionarily^24^ and ecologically^25,26^ important species, producing large “megafaunal” seeds (average of 32 g fresh-mass and 16 g dry-mass per seed) during two to three month mast fruiting events^25,27,28^. As a nationally redlisted (Endangered) species it is illegal to harvest *V. americana* for timber in Brazil^29^. The existing legal regulations are however ambiguous. For example, a recent process to establish 264,500 ha of commercial logging concessions in the Amapá National Forest (a Brazilian sustainable use protected area) states that threatened timber species are “immune” from cutting (i.e. commercial timber harvest of nationally threatened species is not permitted). But at the same time the same process includes *V. americana* as a species for commercial exploitation, with options of 25, 30 or 35 year cutting cycles (http://www.florestal.gov.br/documentos/concessoes-florestais/amapa-licitacao, documents accessed 4 January 2021).Yet, studies from French Guiana. using simulations under more precautionary logging regimes (typical for French Guiana), with large cutting diameter (> 60 cm diameter) and long cutting cycles (65 years), showed *V. americana* would experience unsustainable decreases in both number and basal area^18^. Additionally, monitoring of regeneration/recruitment patterns is not compulsory within Brazilian logging concessions, with the monitoring of forest growth dynamics merely suggested as an optional component.

Fruit-fall patterns are an important part of recruitment and ecosystem services that are not usually included in general projections of biomass and carbon models^30–33^. Although patterns in overall fruit production have been relatively well studied in several Neotropical locations e.g. Cocha Cachu^1^, Barro Colorado^34^ and Nouragues^35^, there remain few fruit-fall data from across the Amazon basin. Mast fruiting is a synchronous and massive production of fruit at long (supra-annual) intervals by plant populations^36,37^. Slow production rates and synchronization can allow masting trees to accumulate carbon, flush massively, and escape from herbivore attacks^37^. However, we still lack an understanding of the ecological factors that determine spatio-temporal variability of seed production in Amazonian masting species such as *V. americana*. Indeed, the contribution of biomass produced (*V. americana* can produce 4000 fruits, representing ≈ 100 kg of fresh mass per tree) has not been evaluated in this rapidly declining species.

The objectives of this study were to: (1) quantify and explain variation in the occurrence and biomass of fallen fruits of *V. americana*, (2) predict meso-scale patterns across 25 km^2^ using topographic, hydrographic, spatial and vegetation cover variables and (3) determine the contribution of the fallen fruits in terms of overall above ground biomass. This modelling study enables us to provide information on the environmental factors that explain and predict meso-scale patterns of fruit-fall biomass and generate new insight into the role of the endangered species *V. americana* in biomass and nutrient cycles in lowland Amazon forest systems.

## Results

### Dominance of* Vouacapoua americana* fruit-fall

**Figure 1:**
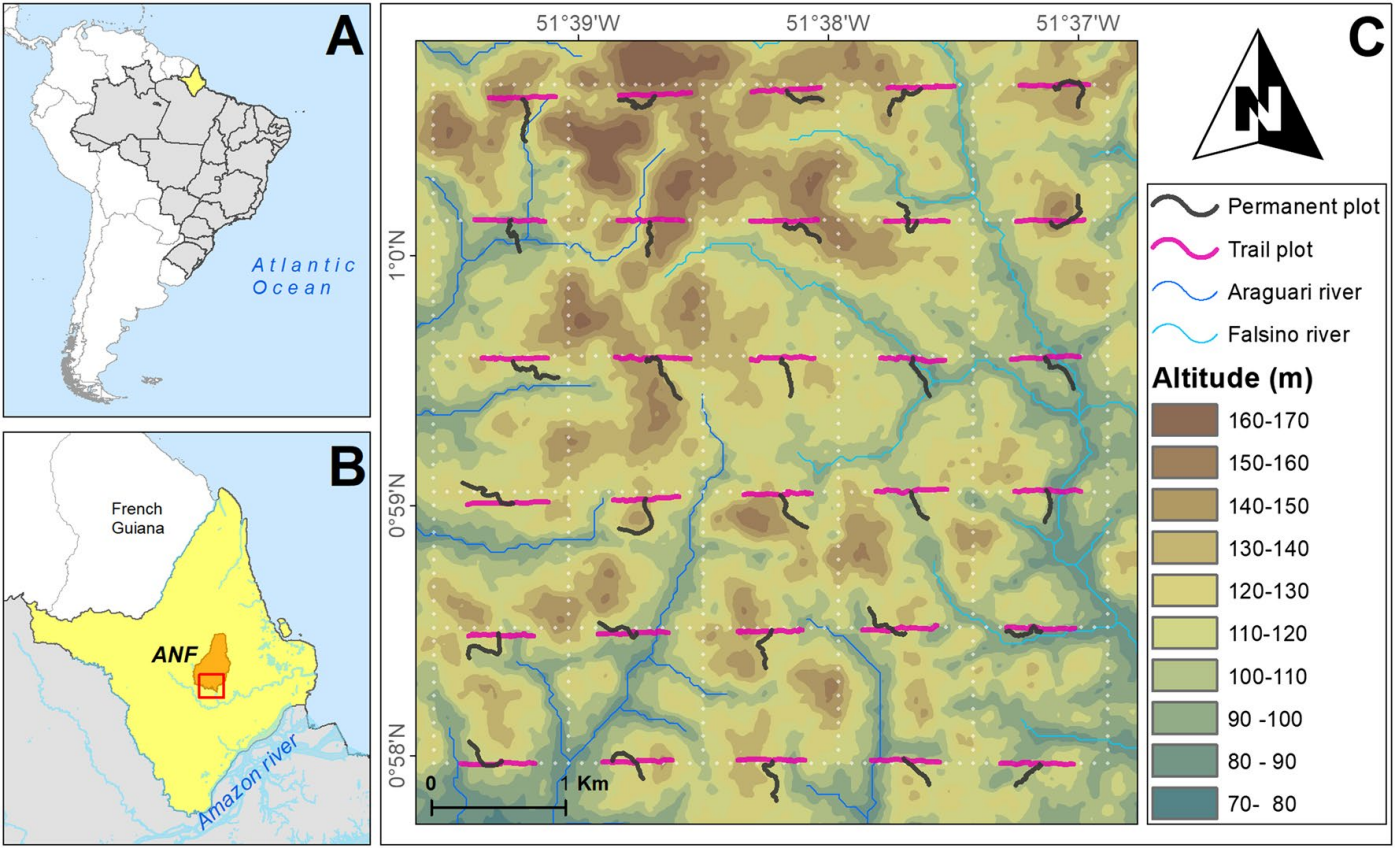
Location of the study site in Amapá National Forest (ANF), Amapá State, north-eastern Brazilian Amazon. (**A**) State of Amapá in Brazil. (**B**) Location of ANF in Amapá. (**C**) Elevation (10-m) across the grid system (white dotted lines); permanent and trail sampling plots (black and pink solid lines respectively) where fruit-fall ground surveys were conducted from May to June 2016. This figure was generated using ArcGIS version 10.8 (https://desktop.arcgis.com/en/arcmap/). Country boundaries and basemaps were obtained from Natural Earth (https://www.naturalearthdata.com/).

A total of 4.4 ha (n = 90) sample plots provided a representative survey across the range of environmental variables within the 25 km^2^ Amapá National Forest sample grid (Fig. 1). Between May and June 2016 fallen fruits were recorded from 87 of the 90 plots (Supplementary Fig. S1). Over these 2 months we counted 21,812 fallen fruits (total dry mass collected 398.3 kg) with a diameter ≥ 1 cm in 4.4 ha, representing 1.8% of the 25 km^2^ ANF grid (Supplementary Table S1). This total included fruits of 86 species from 28 families and 51 genera (Supplementary Table S1). It was possible to identify 81 (94.1%) to species and five (5.9%) to genus (Supplementary Table S1). Fabaceae was the most species rich family with 24.4% of collected species, followed by Sapotaceae (12.7%) and Lecythidaceae (8.1%).

**Figure 2:**
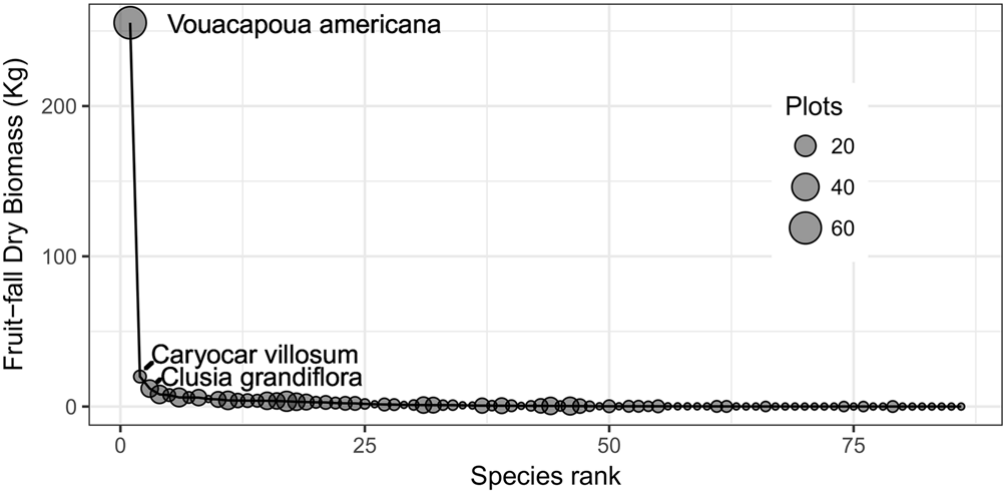
*Vouacapoua americana* fruit-fall dominance. Distribution of species ranked by fruit-fall dry biomass collected in 90 survey plots in the Amapá National Forest from May to June 2016. Size of spheres proportional to the number of plots where each species was recorded.

From May to June 2016 the number and biomass of fallen fruits was dominated by *Vouacapoua americana* (43% and 64%, number and biomass respectively, Fig. 2, Supplementary Table S1). Although *V. americana* was the most dominant and widespread of the fallen fruits (recorded in 62 of 90 plots), there was substantial variation in the number, taxonomic diversity and biomass of fallen fruit between the sampled plots (Table 1, Supplementary Table S1, Supplementary Fig. S1).

**Table 1:** Fruit-fall biomass and taxonomic diversity. Summary statistics of fruit-fall biomass and taxonomic diversity recorded in 90 survey plots in the Amapá National Forest.

Variable	Range	Mean (± SD)
*Vouacapoua americana* fruit-fall dry biomass (kg ha^−1^ month^−1^)	0.0–176.1	28.5 (± 37.4)
Overall fruit-fall dry biomass (kg ha^−1^ month^−1^)	0.0–194.6	44.8 (± 45.1)
Richness (N° Species ha^−1^)	0.0–120.0	44.6 (± 27.3)
Shannon Diversity Index	0.0–1.9	0.8 (± 0.5)

### Meso-scale fruit-fall patterns

**Figure 3:**
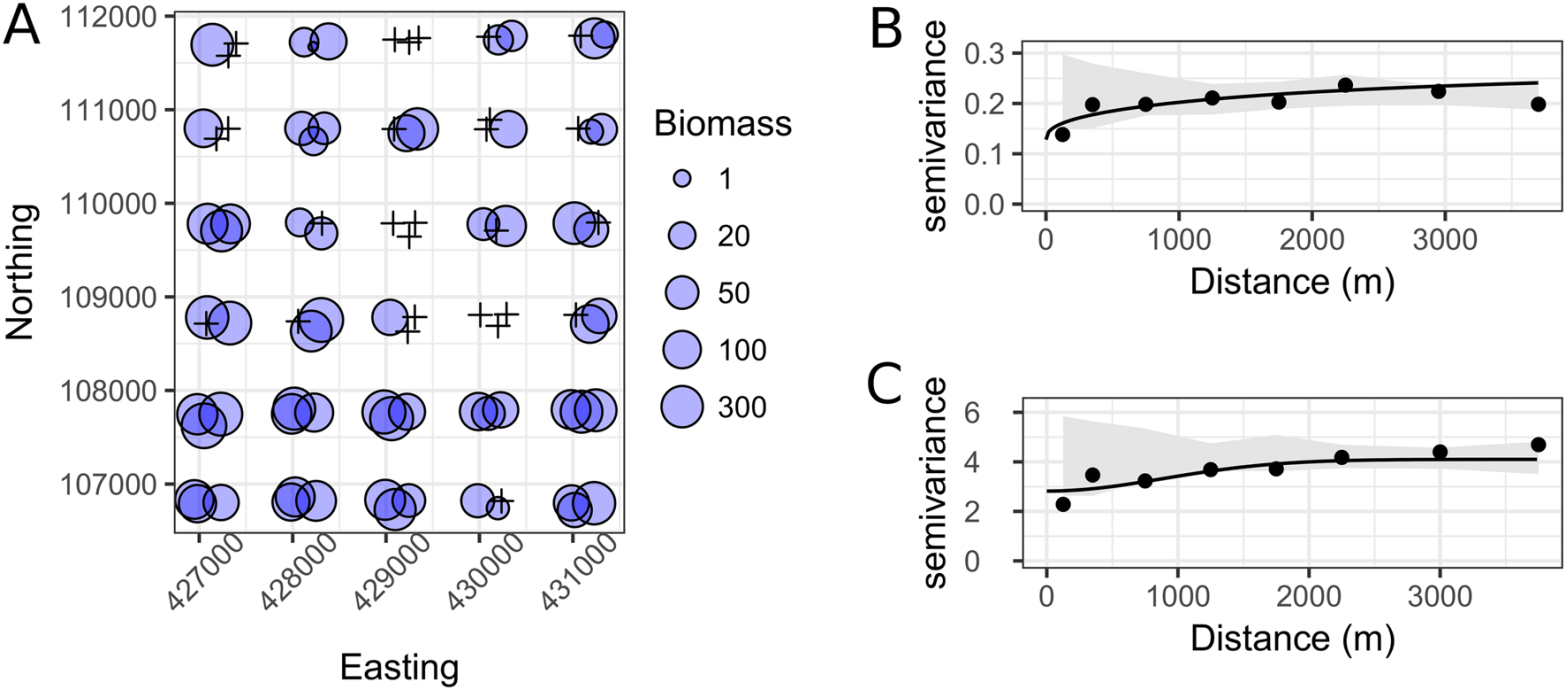
Spatial patterns in *Vouacapoua americana* fruit-fall. (**A**) dry fruit biomass (kg ha^−1^) recorded in 90 survey plots in the Amapá National Forest. Area of blue spheres proportional to biomass and crosses show plots without fallen fruits. Variograms show spatial autocorrelation patterns for biomass (**B**) and presence (**C**) in the 90 plots. Filled points show the estimated empirical variogram values, solid lines are the modelled variogram, and grey shading envelops representing complete spatial randomness obtained by permutation (n = 999 iterations) of the data values.

There was substantially more variation in biomass compared with occurrence of *V. americana* fallen fruits (Fig. 3). Considering the spatial scale of the samples (pairwise distances between plots ranged from 53 to 6642 m), spatial autocorrelation was detected only across relatively short distances (Fig. 3), with complete spatial randomness characterizing the variance in both responses beyond 200 m. The modelled variograms showed relatively high nugget values, representing 50 and 68% of sill values, for fruit-fall occupancy and biomass respectively (Fig. 3).

A combination of spatial, topographic, hydrographic and vegetation cover variables were retained as important for explaining both fruit-fall presence and biomass (Table 2). The spatial model was the most important for explaining patterns in meso-scale masting presence and biomass (Table 2). Topography, hydrography, and vegetation cover models only weakly explained meso-scale biomass patterns (Table 2). In contrast, a relationship with vegetation cover was strongly supported for the meso-scale presence of masting, but topography and hydrography were again only weakly supported. The RMSE of minimal model fitted values (Table 2) was well below the observed SD values for both responses (RMSE = 50.6, 0.35; SD = 74.9, 0.47; biomass, presence respectively).

**Table 2:** Explaining fruit-fall patterns. Models used to explain patterns of *Vouacapoua americana* fruit-fall recorded in 90 survey plots in the Amapá National Forest. (**A**) A priori models and formulas applied to test multiple hypotheses for each response. (**B**) Summary of GAM results for each model and for each response.

**(A)**							
Model	Model	Formula					
1	Space only	Response ~ s(Latitude, Longitude) + Basin					
2	Topography	Response ~ s(Altitude) + s(Slope) + s(Aspect)					
3	Hydrography	Response ~ s(DND) + s(TWI) + (SWI)					
4	Vegetation cover	Response ~ s(AGLB) + s(EVI_diff_) + s(EVI_wet_) + s(EVI_dry_)					
5	Full	Response ~ s(Latitude, Longitude) + s(Altitude) + s(Slope) + s(Aspect) + s(DND) + s(TWI) + s(SWI) + s(AGLB) + s(EVI_diff_) + s(EVI_wet_) + s(EVI_dry_)					

**(B)**							
Response	Model	Var^a^	DE (%)^b^	AIC^c^	BIC^d^	RMSE^e^	Cor^f^
Biomass	Space only	2	50.6	835.7	897.4	51.3	0.73
	Topography	3	6.3	872.1	888.5	71.7	0.28
	Hydrography	3	7.4	868.2	880.7	78.0	0.26
	Vegetation cover	4	5.9	871.0	885.5	71.8	0.26
	Full	12	27.7	829.4	896.2	73.1	0.61
	Minimal	**4**	**56.6**	**827.3**	**895.8**	**50.6**	**0.75**
Minimal Formula: y ~ s(Lat, Long) + s(Slope) + s(DND) + (EVI_diff)							
Presence	Space only	2	23.2	96.0	108.8	0.38	0.55
	Topography	3	11.4	109.0	121.7	0.43	0.38
	Hydrography	3	9.2	111.6	124.4	0.44	0.31
	Vegetation cover	4	25.6	102.0	125.8	0.39	0.52
	Full	12	36.1	87.8	108.5	0.35	0.65
	Minimal	**6**	**36.8**	**87.7**	**109.1**	**0.35**	**0.66**
Minimal Formula: y ~ s(Lat, Long) + Basin + Slope + s(DND) + s(SWI) + AGLB							

Variable acronyms: *TWI* Topographic wetness index, *SWI* SAGA wetness index, *DND* distance to network drainage, *AGLB* above ground live biomass, *EVI* enhanced vegetation index.

^a^Number covariates included in model.

^b^Percentage of Deviance Explained for each model (DE (%)).

^c^Akaike Information Criterion value for each model (AIC).

^d^Bayesian Information Criterion values for each model (BIC).

^e^Root mean square error (RMSE) between observed and fitted model values.

^f^Pearsons correlation between observed and fitted model values from the 90 sample plots.

### Spatial predictions

**Figure 4:**
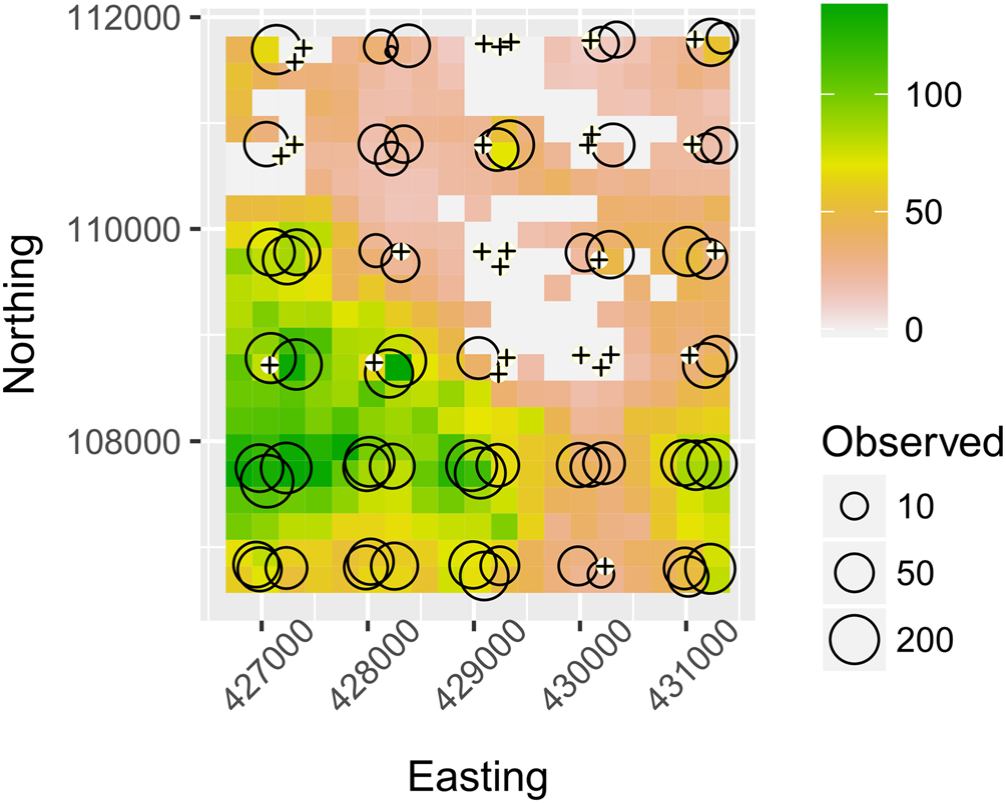
Interpolation of *Vouacapoua americana* fruit-fall. Interpolated biomass across the Amapá National Forest survey grid at 250 m pixel resolution. White spheres with crosses show locations of plots without fallen fruits. Predicted values (color scale, kg ha^−1^ month^−1^) are the weighted mean from six methods (Inverse Distance Weight, Universal Kriging, Generalized Additive Model, Generalized Linear Model, Random Forest and Support Vector Machine. For model details see Supplementary S1).

During the survey period, fallen fruit of *V. americana* were likely to be present in 84.7% (21.1 km^2^) of the survey grid (Fig. 4). Similar to the observed values, the interpolated distribution of fallen *V. americana* fruits was not uniform, with 3 clear clusters of absences (Fig. 4). The spatially explicit interpolations provided an overall dry biomass estimate of 114.7 Mg of fallen fruit during the *V. americana* masting event in the 25 km^2^ area. The above ground live wood biomass for the survey area derived from previously published remote sensing analysis^31,38^ was estimated to be between 694,005 and 1,059,500 Mg. Based on our plot wise monthly mean estimate, we can obtain a maximum value of 538.1 kg ha^−1^ year^−1^ (44.84 kg ha^−1^ month^−1^ × 12). This represents a maximum of 1345 Mg of fallen fruit across the 25 km^2^ area per year. Therefore, annually the fallen fruits represent a maximum of 0.19% of the aboveground biomass of ANF (1345/694,005 Mg). Our spatially explicit models show that the total masting fruit fall biomass from a single species (*V. americana*) represents the rapid transfer of between 0.01 and 0.02% and of the overall above ground standing biomass in the area.

## Discussion

Despite the promotion of sustainable use of timber and non-timber resources in tropical forests, the current management criteria still typically lack inclusion of species-specific ecological features. We were able to model the meso-scale distribution of fallen fruit biomass from the critically endangered *V. americana*. The models enable us to describe, explain and predict meso-scale fruit-fall biomass patterns across 25 km^2^ of lowland Amazon forest. We found that spatial effects most strongly explained variation in fruit-fall patterns and that the contribution of spatial, topographic, hydrographic and vegetation variables differed between responses. We discuss these findings in relation to what is known regarding fruit-fall patterns across lowland Amazonia and then consider the implications for understanding patterns in biomass below the forest canopy.

Our sample provides a representative snapshot of fruit-fall patterns across the 25 km^2^ study area. The composition of families and species follows the general pattern found in nearby forest sites and across the Guiana shield^39–44^. We found fallen fruits from four (*Crhysophyllum*, *Licania*, *Protium* and *Eschweilera*) of the five most abundant genera that Pereira, et al.^43^ identified in 1.9 ha of nearby (32 km distant) lowland terrra-firme forest. Fabaceae was also the dominant family in a recent inventory of large (> 40 cm DBH) trees, close (< 30 km) to our study area^39^.

Fruit-fall biomass was similar to values reported by studies from other regions of the Guianan Shield. In the most productive month in the rainy season, Sabatier^44^ reported a mean of 50 kg ha^−1^ of fruit-fall production, with 86% of species producing fruits during this season. The less productive soils of the Guianan Shield result in lower values of arboreal species richness^2,45^. This pattern also appears to be reflected in fruit production^46^, with values from French Guyana (292 kg ha^−1^ annual fruit-fall dry biomass) less than half those reported from western Amazonia (e.g. Hanya, et al.^46^ recorded 796 kg ha^−1^ of annual fruit-fall dry biomass in Cosha Cashu, Peru).

Clearly *V. americana* was the main source of fruit-fall, in our study site in 2016. From 20 fruits (representative sample of 10 mature fruits from 2 different trees), we obtained a mean dry mass of 18.2 g, the majority of which was the single seed (90%, mean dry weight from 20 seeds = 16.2 g). This provides an estimate of 72.8 kg (4000 × 18.2 g) of dry fruit per tree. Our dry fallen fruit biomass values therefore represent a range of 1 to 5 fruiting trees per Ha. Our aim was not to estimate tree density, but as these values fall within the expected range reported by previous studies they do reinforce the representativeness of our meso-scale sample. There is an urgent need for additional studies to establish the density and distribution of *V. americana* in the study area. This data could then enable our model predictions to be validated.

**Figure 5:**
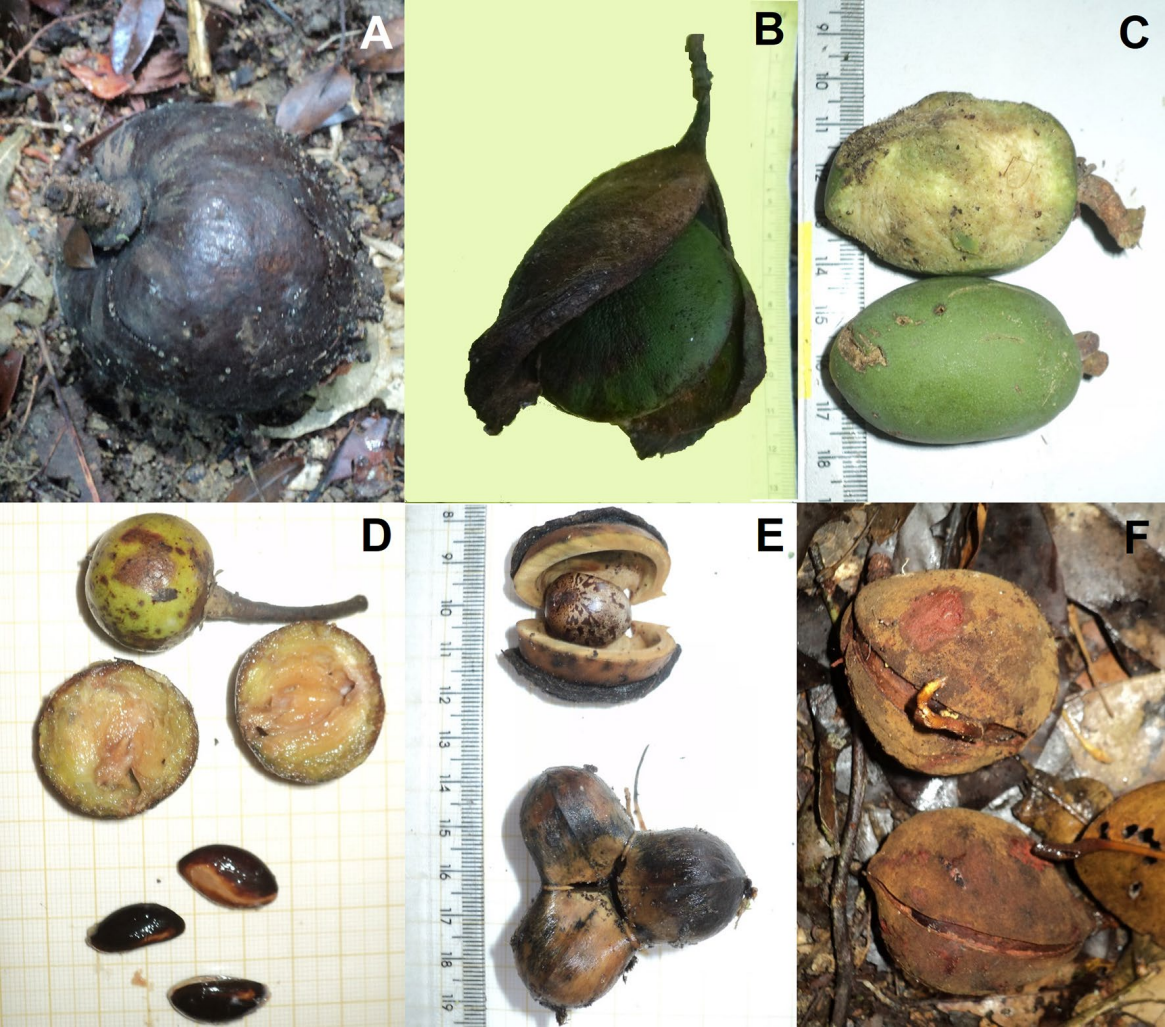
Examples of fruit-fall survey inclusion criteria. Not collected and not counted: (**A**) *Fusaea longifolia* (Annonaceae) rotten fruit: (**B**) *Vatairea guianensis* (Fabaceae) dried fruit. Counted: (**C**) *Dipteryx odorata* (Fabaceae) partially eaten and fresh counted not collected (partially eaten fruit was just counted); (**D**) *Manilkara huberi* (Sapotaceae) collected and counted, fresh fruits and seed collected to weigh; (**E**) *Hevea brasiliensis* (Euphorbiaceae) counted and removed to avoid repetitive sampling; (**F**) *Vouacapoua americana* (Fabaceae) counted and removed. Germinating fruits not collected for weighing and also not counted in a second sampling.

Predicting across a continuous gird enables a variety of analyses that are not possible with sparsely sampled data. Several studies have used remote sensing data to create accurate models of predictions of tropical forest carbon or biomass in diverse scales^30,33,47–52^, but such approaches have not been applied to fruit-fall biomass. The maps presented (Fig. 5) are useful for visualizing the environmental space in more than one dimension and for understanding the predicted responses in the 25 km^2^ study area. These maps provide a baseline reference that enables evaluation of future management and/or silvicultural actions.

Our model predictions show that field data collection will be necessary to generate robust estimates of fruit-fall biomass and enable evaluation of future management and/or silvicultural actions. These estimates will improve our knowledge about what is being actually conserved and where we can find it within the protected area. However, comparison with fruit-fall patterns in other lowland sites is necessary to enable more rigorous model testing and evaluation. As the spatial structure described may also change significantly over time e.g. plots with low fruit-fall in 2016 may be plots with high fruiting in the following years, additional studies are also required to establish the multi-year patterns of fruit-fall dynamics in the study area.

We found that remotely sensed data provided useful environmental explanatory variables for modelling the distribution of fruit-fall dry biomass. More generally, this study shows remotely sensed variables have potential for predicting meso-scale fruit-fall biomass. More studies are necessary to improve predictive power of biomass models for understanding impacts of compositional changes driven by anthropogenic and climate changes.

## Materials and methods

### Ethics statement

Fieldwork and data collection was conducted under research permit numbers IBAMA/SISBIO 40355-1 and 47859-2 to DN, issued by the Brazilian Instituto Chico Mendes de Conservação da Biodiversidade (ICMBio).

### Study area

We sampled fallen fruits at a Brazilian Program for Biodiversity Research (“Programa de Pesquisa em Biodiversidade”—hereafter PPBio^53^) research grid (25 km^2^) in the Amapá National Forest (Floresta Nacional do Amapá—hereafter ANF). ANF is a sustainable-use protected area of approximately of 412,000 ha, centered in the state of Amapá, in north-eastern Brazilian Amazon between the Falsino and Araguari rivers (0° 55′ 29″ N, 51° 35′ 45″ W, Fig. 1).

The regional climate is classified by Köppen–Geiger as Am (Equatorial monsoon)^54^, with an annual rainfall greater than 2000 mm (2240–2510 mm per year from 2010 to 2016)^55^. The driest months are September to November (total monthly rainfall < 150 mm) and the wettest months from February to April (total monthly rainfall > 300 mm)^55^ (Supplementary Fig. S2). The ANF is located within the Uatumã-Trombetas moist forest ecoregion (tropical and subtropical moist broadleaf forests biome) and consists of continuous tropical rainforest vegetation, predominantly never-flooded closed canopy “terra firme” forest^56^. Canopy trees within the ANF typically reach a height of 25–35 m interspersed with emergent trees reaching up to 50-m^56^. The soil is predominantly low-fertility oxisols, including a mix of red, yellow and red-yellow latosols following the Brazilian soil classification system^56,57^.

### Study species

*Vouacapoua americana* is a Critically Endangered (A1cd + 2cd^23^) endemic to the eastern Guiana shield rainforests^24,58^. Guiana Shield forests are highly biodiverse, with a more complicated history than temperate and boreal forests, due to a mixture of gradual compositional changes and expansion from refugia^45,59^. *V. americana* may be a typical example of this situation, as it shows population contraction in its hypothesized range^24,58^. *V. americana* is a large canopy tree with a wide crown that can bear 3000–4000 large (32.6 ± 1.8 g^27^) single-seeded fruits. It is highly valued for its durable hardwood timber (heartwood density 0.91 g/m^3^ at 12% moisture content) and pharmacological potential^60,61^. The density of individuals over 10 cm diameter at breast height (d.b.h.) is of the order of 10 per hectare^59,62,63^, but varies widely with adults showing a locally clustered distribution^65^. The spatial distribution of adults depends on abiotic and biotic conditions, including topography and soil hydromorphy, light^63–66^ and the availability of short and long range dispersal agents^27,63,64^.

Long-term studies from French Guiana show *V. americana* is a mast-seeding species [defined as bimodal seed production with no overlap between two tails^36^] with an average interval between masting events ranging from 2.3^66^ to 4 years^44^. Fruiting occurs during a relatively rapid (typically two month) masting event that is synchronized with the wet season^27,66^. Immature fruits are consumed by primates (including *Ateles* spp. and *Alouatta* spp., http://vision.psychol.cam.ac.uk/spectra/guiana/fdiet.html). Fallen seeds may germinate underneath parent trees but this abiotic mode of recruitment is generally unsuccessful for *V. americana*^27,28,66^ unless there is a canopy opening nearby^28^. Intense removal of *V. americana* seeds leads to high rates of seed dispersal compared with low predation throughout the season^27^. Most seeds are dispersed approximately 10 m away by rodents (*Dasyprocta leporina* and *Myoprocta exilis*)^27,63,65^, with tapir and peccaries thought to be responsible for longer range dispersal^26^.

### Fruit-fall data collection

Fruit-fall ground surveys are a well-established, relatively efficient and straight forward method to assess fruit production in tropical forests and can reflect seasonal fruiting phenology well^5,67^. For example, fruit availability, estimated by fruit-fall, positively affected the biomass and the number of species among frugivorous primates^46,68^. As part of a wider study^69,70^ fruit-fall surveys were conducted in May and June 2016 that coincided with the fruit-fall of *V. americana*. Although we did not examine phenological patterns, based on the findings from previous long-term studies^44,63,64^ we assume that this was a masting fruit-fall event for *V. americana*.

Within the 25 km^2^ PPBio grid, a total of 30 regularly spaced (1-km interval) points were sampled (Fig. 1)^71^. This regular arrangement and sample size of 30 has been shown to be adequate for capturing variation in meso-scale species diversity responses across lowland Amazonia^72^. At each of the 30 sample points, surveys were conducted along three plots (one permanent plot and two trail plots, Fig. 1). The permanent plots (250 m long) are nonlinear and follow altitudinal contours to minimize the internal variation in both altitude and correlated covariates such as soil type^53,71^. Additionally, we sampled two linear trail plots (250 m each), one before and the other after the start point of the permanent plots (Fig. 1). Fallen fruit were sampled 1 m to each side of the plot centerlines i.e. covering a total area of 500 m^2^ (2 × 250 m) for the linear trail plots. This effort provided a total sampled area of 4.42 ha (plot area m^2^ mean ± SD = 490.7 ± 46.9).

To obtain robust and reproducible estimates of fresh fallen fruit we established a number of inclusion and exclusion criteria^6^ (Fig. 5). All fresh (i.e. not rotten or desiccated) fallen fruits were counted. Fruits considered unlikely to change in appearance between samples (i.e. *Vouacpoua americana*, *Apeiba sp*, *Hevea brasiliensis*) were removed in order to avoid counting the same fruit twice. Fresh fruits that had been partially eaten were also counted (Fig. 5).

The collected fruits were identified to the lowest taxonomic level (Supplementary Fig. S3) and named following APG III^73^ by botanists from the Amapá State Scientific Research and Technology Institute (Instituto de Pesquisas Científicas e Tecnológicas do Estado do Amapá, IEPA). To obtain dry fruit biomass estimates the mean dry weight from a maximum of 30 mature fruits of all fallen fruit species was calculated (Supplementary Table S1). Fruits with seeds beginning to germinate were not weighed. The collected fruits were dried to constant weight in an oven at 50 °C and then weighed with a precision balance (fruits < 10 g) or digital balance with error ± 0.01 g (fruits > 10 g). Values of fruit-fall dry biomass were expressed as kg ha^−1^.

### Environmental explanatory variables

Remote sensing data represent continuous measurements of environmental variables that can be applied in ecological studies^69,72,74,75^. To explain and predict patterns in fallen fruit we used a total of 10 remotely sensed variables (Table 3). These remotely sensed variables were selected to represent meso-scale patterns in topography, hydrography and vegetation cover (Table 3, Supplementary Fig. S4).

**Table 3:** Explanatory variables. Summary statistics of variables used to explain patterns in *Vouacpoua americana* fruit-fall.

Model	Variable^a^ (units)	Ecological relevance	Source^b^	Grid^c^	Plot values		Study area values	
					Mean (SD)	Range	Mean (SD)	Range
Topography	Altitude (m)	Proxy for edaphic conditions that affect productivity	SRTM	92	120 (12.6)	94–154.7	117.2 (14.5)	67.0–182.0
	Slope (%)	Proxy for edaphic conditions	SRTM	92	7.6 (2.6)	1.9–16.7	7.7 (3.1)	1.3–19.1
	Aspect (Degree)	Proxy for differences in exposure to prevailing winds	SRTM	92	181.8 (65.3)	53.1–337.6	186.9 (69.2)	21.1–347.1
Hydrography	TWI (None)	Proxy for hydrographic conditions that affect occurrence	SRTM	92	13.5 (2.0)	8.3–18.7	13.7 (4)	2.3–23.6
	SWI (None)	Proxy for hydrographic conditions that affect occurrence	SRTM	92	7.5 (0.8)	5.3–9.2	7549 (0.9)	4647–11,029
	DND (m)	Proxy for hydrographic conditions that affect occurrence. Obtained from interaction between HAND and HDND	SRTM	92	8375 (8391)	8–46,975	7523 (7125)	81–46,908
Vegetation cover	AGLB (Mgha^−1^)	Masting influenced by resource availability, proxy for productivity	MODIS/LiDAR^d^	30	281.8 (25.8)	203.0–322.5	277.1 (30.3)	31.0–328.8
	EVI^e^ wet (− 1 to 1)	To represent vegetation (tree canopy) changes, and as a proxy of leaf area and photosynthetic capacity. Means calculated from values during wet season (3 months prior to sampling), preceding dry season (September to November 2015) and the difference between the mean season values	MODIS	250	0.49 (0.03)	0.41–0.58	0.49 (0.05)	0.37–0.71
	EVI^e^ dry (− 1 to 1)		MODIS	250	0.60 (0.03)	0.52–0.69	0.59 (0.04)	0.43–0.78
	EVI^e^ diff (− 1 to 1)		MODIS	250	0.11 (0.04)	0.01–0.19	0.10 (0.05)	− 0.16–0.30

^a^Variable acronyms: TWI (Topographic wetness index), SWI (SAGA wetness index), DND (Distance to Network Drainage), HAND (Height above network drainage), HDND (Horizontal distance to network drainage), AGLB (Above ground live biomass), EVI (Enhanced Vegetation Index).

^b^Remote sensed data source.

^c^Native cell resolution (meters) of source raster grid.

^d^Derived from MODIS, imagery using GLAS (Geoscience Laser Altimeter System) LiDAR data, see Baccini et al.^31^ for technical details.

^e^Enhanced Vegetation Index^76,77^. Area with less leaf area (less green) during the preceding dry season could have increased flowering and therefore increased masting fruit-fall in the wet season.

### Model development

Complementary approaches were adopted to explain and predict patterns in fruit-fall presence and biomass. Firstly, to examine spatial patterns in the responses we used variograms that enable quantification of different aspects of the spatial patterns, including: range (the limit of spatial dependence), nugget (portion of semi-variance attributable to random/environmental factors), and sill (distance at which the variogram becomes constant)^78^. Then, to examine the environmental factors explaining patterns of fruit-fall presence and dry biomass Generalized Additive Models (GAMs) were employed^79,80^. GAMs are a nonparametric extension of general linear models that provide the flexibility to model non-parametric and nonlinear relationships that are typical of many ecological patterns. Explanatory GAMs were generated using the “mgcv” package^81^ using R 3.10 software^82^. Penalized cubic regression splines determined the shape of nonparametric functions, with the degree of smoothing selected automatically via maximum likelihood using the mgcv package defaults for all models.

To avoid subjective bias in model development we did not examine correlation among variables until after formulation of our a priori models. We applied a two stage approach to explain patterns in the fruit-fall responses. Firstly, separate models were developed to examine the effects of space, topography, hydrography and vegetation cover (Table 2). Within each of our four models, explanatory variables were tested for collinearity. The explanatory power of the models and the level of support for the different hypotheses were examined within a maximum likelihood framework^83^, with models tested and compared using a combination of model deviance explained^81^ and information criteria (AIC and BIC)^83^. The accuracy of model fits were examined using the root mean square error (RMSE) and correlations between observed and fitted values.

Secondly, we obtained the most parsimonious (“minimal”) model, with the most important variables selected using a manual backward stepwise selection based on minimizing AIC values. Manual selection started from the full model without correlated variables, with stepwise exclusion of variables if they did not make a statistically significant (p ≥ 0.05) contribution.

After explaining variation in responses, we then used an ensemble model to predict the presence and dry biomass of masting fruit-fall across the 25 km^2^ sample grid. The similarity in environmental variable values between the 90 plots and the 25 km^2^ grid (Table 3) means that we consider the predictions to be an interpolation within the range of environmental values. Ensemble approaches were used to decrease the predictive uncertainty of single-models by combining their projections. The ensemble model was obtained from the weighted mean of six single model methods. The six model algorithms were inverse distance weighted (IDW^84^), universal kriging (UK^84^), generalized linear model (GLM), generalized additive model (GAM^80^), random forest (RF^85^) and support vector machine (SV^86^). Predictive models were developed for both presence and biomass with the weighted mean ensemble derived from model predictions weighted by the correlation between observed and predicted values.

Finally, we compared the predicted values with overall live wood biomass. We estimated the range of overall live wood biomass in the 25 km^2^ study area from two sources^31,38^. The 500 m resolution mapped values from Baccini et al.^31^ were used to provide minimum value and the maximum value was estimated by extrapolating the mean (423.8 Mg ha) from Johnson et al.^38^ across the 25 km^2^ (2500 ha) area.

## Supplementary Information

Supplementary Information.

## Data Availability

The datasets generated during and/or analyzed during the current study are available from the corresponding author on reasonable request.
