# Supplementary material for: Contribution of Vouacapoua americana fruit-fall to the release of biomass in a lowland Amazon forest
Source: Sci Rep. 2021 Feb 22;11:4302. doi: 10.1038/s41598-021-83803-y (PMC7900201; doi:10.1038/s41598-021-83803-y)

**Contribution of *Vouacapoua americana* fruit-fall to the release of biomass in a lowland Amazon forest**

Victor Juan Ulises Rodriguez-Chuma^1^ and Darren Norris^1,2*^

**Table S1. Biomass, abundance and frequency of fallen fruits species found in in Amapá National Forest.** Abundance defined as the number total of records. Frequency defined as number of plots where species were recorded.

| **Family/Species** | **Dry mass (g)** | **Dry mass fruit (%)** | **Abundance** | **Frequency** |
| --- | --- | --- | --- | --- |
| **ANNONACEAE** |  |  |  |  |
| *Duguetia cadaverica* Huber. | 26.5 | 0.007 | 2 | 1 |
| *Fusaea longifolia* (Aubl.) Saff. | 468.7 | 0.118 | 11 | 6 |
| **APOCYNACEAE** |  |  |  |  |
| *Ambelania acida* Aubl. | 35.6 | 0.009 | 10 | 1 |
| *Geissospermum* laeve (Vell.) Miers. | 229.5 | 0.058 | 9 | 2 |
| *Geissospermum* sericeum Miers. | 3205.3 | 0.805 | 1027 | 11 |
| **ARECACEAE** |  |  |  |  |
| *Astrocaryum gynacanthum* Mart. | 4.2 | 0.001 | 1 | 1 |
| *Euterpe oleracea* Mart. | 2339.7 | 0.587 | 1786 | 3 |
| **BIGNONIACEAE** |  |  |  |  |
| *Fridericia mollis* (Vahl) L.G.Lohmann. | 30.8 | 0.008 | 1 | 1 |
| *Jacaranda copaia* (Aubl.) D.Don. | 7619.7 | 1.913 | 109 | 4 |
| **BOMBACACEAE** |  |  |  |  |
| *Pachira* sp. | 30.2 | 0.008 | 3 | 1 |
| **BURSERACEAE** |  |  |  |  |
| *Protium* sp1. | 420.5 | 0.106 | 378 | 11 |
| *Protium* sp2. | 321.7 | 0.081 | 383 | 12 |
| *Protium* *tenuifolium* (Engl.) Engl. | 180.9 | 0.045 | 86 | 4 |
| *Tetragastris* *altissima* (Aubl.) Swart. | 201.7 | 0.051 | 80 | 4 |
| **CARYOCARACEAE** |  |  |  |  |
| *Caryocar* *glabrum* (Aubl.) Pers. | 2100.7 | 0.527 | 33 | 5 |
| *Caryocar* *villosum* (Aubl.) Pres. | 19972.7 | 5.015 | 151 | 4 |
| **CHRYSOBALANACEAE** |  |  |  |  |
| *Licania* *canescens.* | 470.9 | 0.118 | 75 | 2 |
| *Licania* *macrophylla* Benth. | 139.5 | 0.035 | 3 | 1 |
| *Licania* sp1. | 6069.9 | 1.524 | 263 | 14 |
| *Licania* sp2. | 44.3 | 0.011 | 7 | 2 |
| **CLUSIACEAE** |  |  |  |  |
| *Clusia grandiflora* Splitg. | 11969.8 | 3.005 | 282 | 11 |
| **EBENACEAE** |  |  |  |  |
| *Diospyros dichroa* Sandwith. | 2994.3 | 0.752 | 177 | 8 |
| **ELAEOCARPACEAE** |  |  |  |  |
| *Sloanea garckeana* K.Schum.. | 525.3 | 0.132 | 270 | 3 |
| *Sloanea grandiflora* Sm. | 697.22 | 0.175 | 126 | 1 |
| *Sloanea* sp. | 16.3 | 0.004 | 2 | 1 |
| **EUPHORBIACEAE** |  |  |  |  |
| *Glycidendron* sp. | 5040.0 | 1.265 | 280 | 1 |
| *Hevea* *brasiliensis* (Willd. ex A. Juss.) Müll. Arg. | 4019.9 | 1.009 | 407 | 6 |
| *Mabea* *caudata* Pax & K.Hoffm. | 37.3 | 0.009 | 44 | 1 |
| **FABACEAE** |  |  |  |  |
| *Batesia* *floribunda* Benth. | 32.1 | 0.008 | 7 | 1 |
| *Cassia* *fastuosa* Willd. ex Benth. | 157.2 | 0.039 | 3 | 1 |
| *Dinizia* *excelsa* Ducke. | 181.3 | 0.046 | 35 | 2 |
| *Dipteryx odorata* (Aubl.) Willd. | 955.3 | 0.240 | 77 | 8 |
| *Hymenaea* *courbaril* L. | 1558.5 | 0.391 | 56 | 1 |
| Indet. 2. | 615.0 | 0.154 | 249 | 2 |
| *Inga* *alba* (Sw.) Willd. | 3878.5 | 0.974 | 109 | 4 |
| *Inga* *capitata* Desv. | 8069.4 | 2.026 | 407 | 13 |
| *Inga* *heterophylla* Willd. | 7.9 | 0.002 | 1 | 1 |
| *Inga* sp1. | 1373.0 | 0.345 | 102 | 4 |
| *Inga* sp2. | 191.2 | 0.048 | 15 | 1 |
| *Inga* sp3. | 937.2 | 0.235 | 208 | 2 |
| *Inga* sp4. | 475.0 | 0.119 | 32 | 1 |
| *Inga* sp5. | 186.3 | 0.047 | 17 | 3 |
| *Inga* sp6. | 17.8 | 0.004 | 2 | 1 |
| *Inga* sp7. | 39.3 | 0.010 | 8 | 1 |
| *Mucuna* sp. | 2826.8 | 0.710 | 22 | 3 |
| *Parkia* *pendula* (Willd.) Walp. | 29.0 | 0.007 | 7 | 1 |
| *Pseudopiptadenia* *psilostachya* (DC.) G.P.Lewis & M.P.Lima. | 6050.6 | 1.519 | 193 | 3 |
| *Vatairea* *guianensis* Aubl. | 3831.6 | 0.962 | 255 | 11 |
| *Vouacapoua* *americana* Aubl. | 255488.5 | 64.146 | 9425 | 62 |
| **HUMIRIACEAE** |  |  |  |  |
| *Vantanea* sp. | 1138.0 | 0.286 | 26 | 1 |
| **UNIDENTIFIED** |  |  |  |  |
| Indet 1. | 85.3 | 0.021 | 1 | 1 |
| Indet 3. | 50.4 | 0.013 | 13 | 1 |
| Indet 4. | 155.2 | 0.039 | 40 | 1 |
| Indet 5. | 211.6 | 0.053 | 65 | 1 |
| **LECYTHIDACEAE** |  |  |  |  |
| *Couratari* *guianensis* Aubl. | 3948.7 | 0.991 | 84 | 5 |
| *Couratari* *multiflora* (Sm.) Eyma. | 117.8 | 0.030 | 104 | 3 |
| *Eschweilera* *alata* A.C.Sm. | 2153.1 | 0.541 | 95 | 5 |
| *Eschweilera* *pedicellata* (Rich.) S.A.Mori. | 4752.4 | 1.193 | 228 | 8 |
| *Eschweilera* sp1. | 1742.7 | 0.438 | 53 | 2 |
| *Eschweilera* sp2. | 1287.0 | 0.323 | 76 | 3 |
| *Lecythis* *corrugata* Poit. | 1003.7 | 0.252 | 211 | 9 |
| **LOGAGINACEAE** |  |  |  |  |
| *Strychnos* sp. | 176.3 | 0.044 | 2 | 1 |
| **MELASTOMATACEAE** |  |  |  |  |
| *Bellucia* *grossularioides* (L.) Triana. | 10.5 | 0.003 | 16 | 1 |
| **MELIACEAE** |  |  |  |  |
| *Carapa* *guianensis* Aubl. | 118.8 | 0.030 | 2 | 1 |
| **MORACEAE** |  |  |  |  |
| *Ficus* sp | 49.4 | 0.012 | 12 | 1 |
| *Helicostylis* *pedunculata* Benoist | 17.0 | 0.004 | 19 | 3 |
| **MYRTACEAE** |  |  |  |  |
| *Eugenia* sp. | 557.7 | 0.140 | 303 | 8 |
| *Psidium* *sartorianum* (O.Berg) Nied. | 24.0 | 0.006 | 68 | 2 |
| **OCHNACEAE** |  |  |  |  |
| *Ouratea* sp. | 15.7 | 0.004 | 12 | 1 |
| **PASSIFLORACEAE** |  |  |  |  |
| *Passiflora* sp. | 86.8 | 0.022 | 30 | 3 |
| **POLYGALACEAE** |  |  |  |  |
| *Moutabea* guianensis | 850.0 | 0.213 | 50 | 1 |
| **SAPINDACEAE** |  |  |  |  |
| *Paullinia* sp. | 16.7 | 0.004 | 100 | 1 |
| **SAPOTACEAE** |  |  |  |  |
| *Chrysophyllum* *durifructum* (W.A.Rodrigues) T.D.Penn. | 1048.3 | 0.263 | 18 | 2 |
| *Manilkara* *huberi* (Ducke) Chevalier. | 3494.0 | 0.877 | 1008 | 17 |
| *Manilkara* sp1. | 10.4 | 0.003 | 15 | 1 |
| *Manilkara* sp2. | 188.0 | 0.047 | 94 | 3 |
| *Pouteria* *heterosepala.* | 23.6 | 0.006 | 2 | 1 |
| *Pouteria* *hispida* Eyma. | 320.5 | 0.080 | 157 | 6 |
| *Pouteria* *jariensis* Pires & T.D.Penn. | 27.3 | 0.007 | 11 | 2 |
| *Pouteria* *macrophylla* (Lam.) Eyma. | 5958.2 | 1.496 | 82 | 9 |
| *Pouteria* sp1. | 2746.5 | 0.690 | 146 | 5 |
| *Pouteria* sp2. | 943.8 | 0.237 | 46 | 2 |
| *Pouteria* torta. | 386.4 | 0.097 | 23 | 2 |
| **STERCULIACEAE** |  |  |  |  |
| *Sterculia* *excelsa* Mart. | 4105.7 | 1.031 | 226 | 13 |
| **TILIACEAE** |  |  |  |  |
| *Apeiba* *macropetala* Ducke. | 3734.0 | 0.938 | 310 | 9 |
| **VOCHYSIACEAE** |  |  |  |  |
| *Vochysia* *rufescens* W.A.Rodrigues | 619.4 | 0.156 | 834 | 7 |

**Figure S1. Spatial distribution of response variables.**


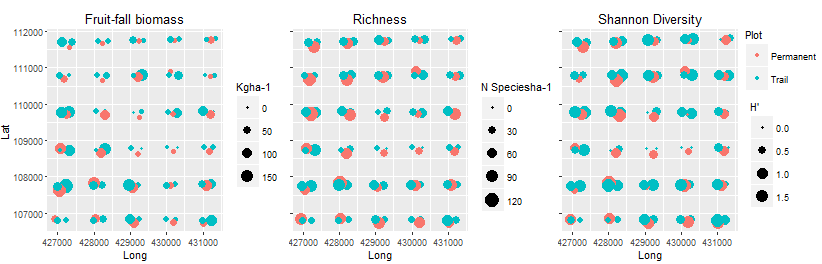


**Figure S2: Study area rainfall**

Fallen fruits were collected during the end of the wet season (May – June 2016, Fig. S1). During the survey period total monthly rainfall was 310 and 219 mm (monthly totals for May and June respectively).

**
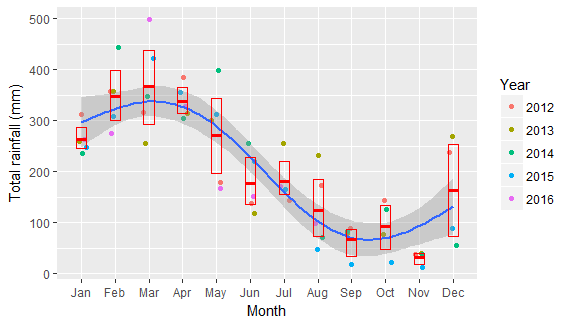
**

Figure S2. Monthly rainfall recorded close (36 km) to the Amapá National Forest study site. Weather station data available from the Brazilian National Water Agency (station ID: 8052000, ANA 2016). Monthly totals are presented from five years (2012, 2013, 2014, 2015 and 2016). Boxplots show means and 95% confidence limits estimated via nonparametric bootstrap. The blue line and shaded areas are the mean value and 95% confidence intervals from a GAM model illustrating the trend in rainfall.

**References**

ANA. Sistema de Monitoramento Hidrológico (Hydrological Monitoring System). Agência Nacional de Águas[[nl]]National Water Agency, Available at <http://www.snirh.gov.br/hidroweb/publico/medicoes_historicas_abas.jsf>. 2018 [20.07.2018].

**Figure S3. Field guide of fruits found in Amapá National Forest**. Complete version available at: <https://www.researchgate.net/publication/312318151_Fruits_of_Amapa_National_Forest>


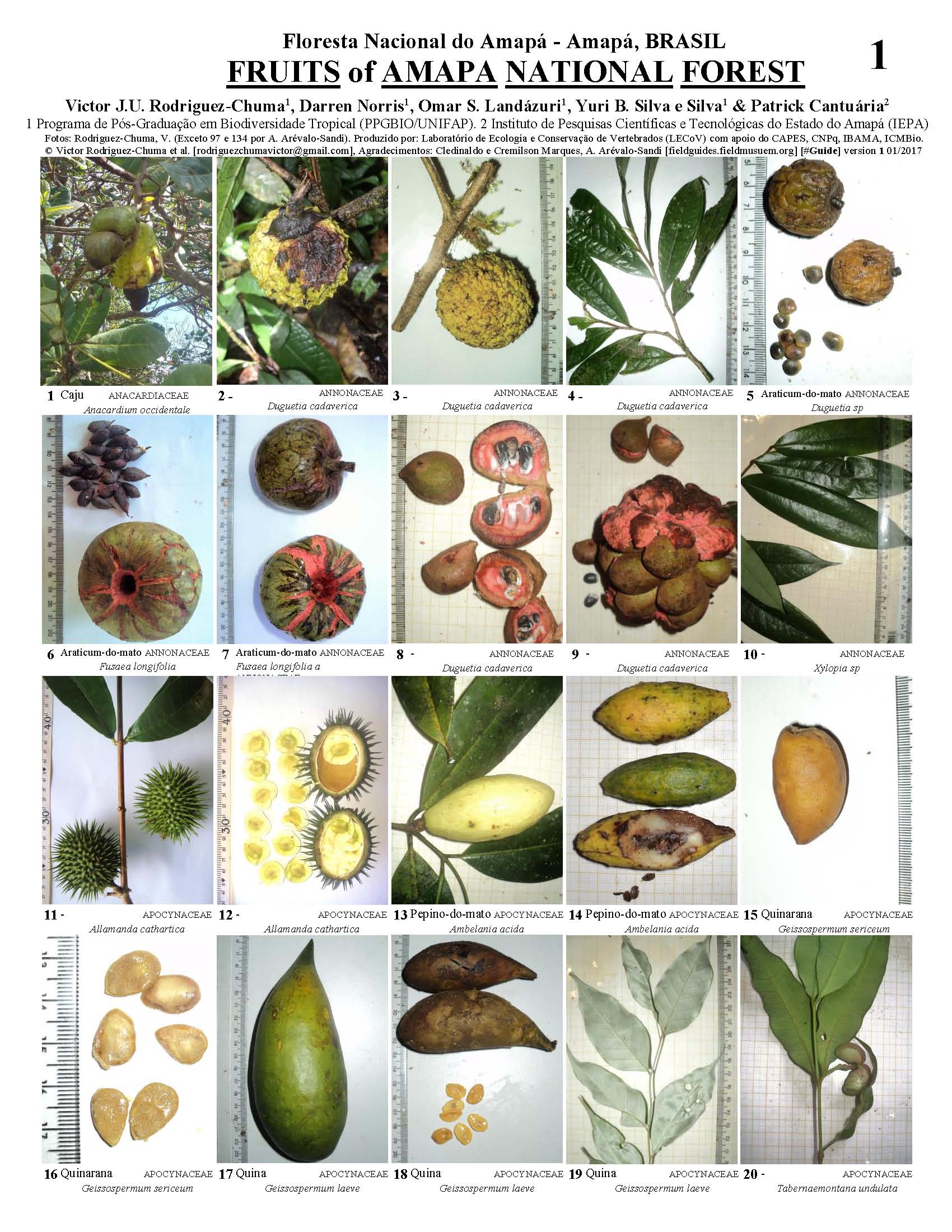


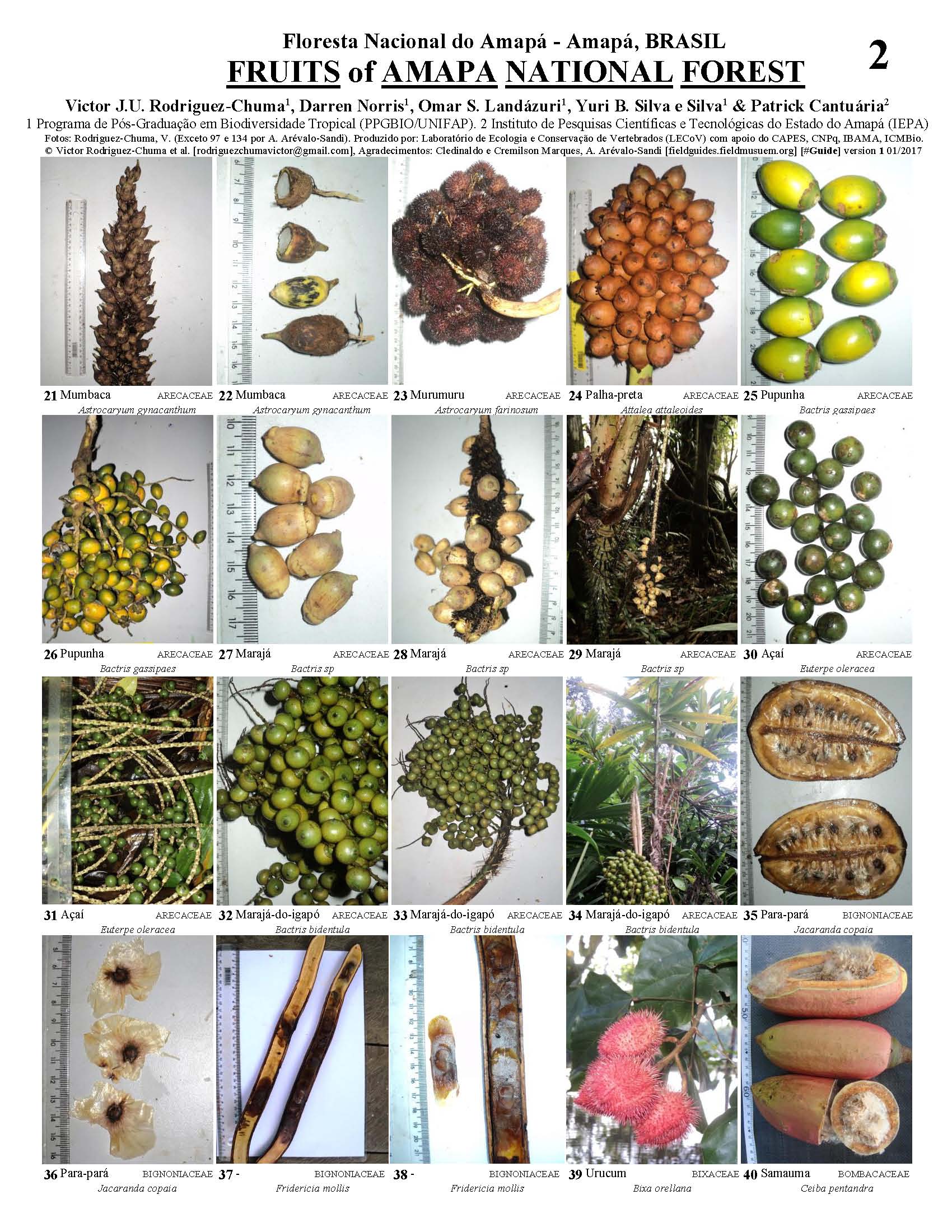


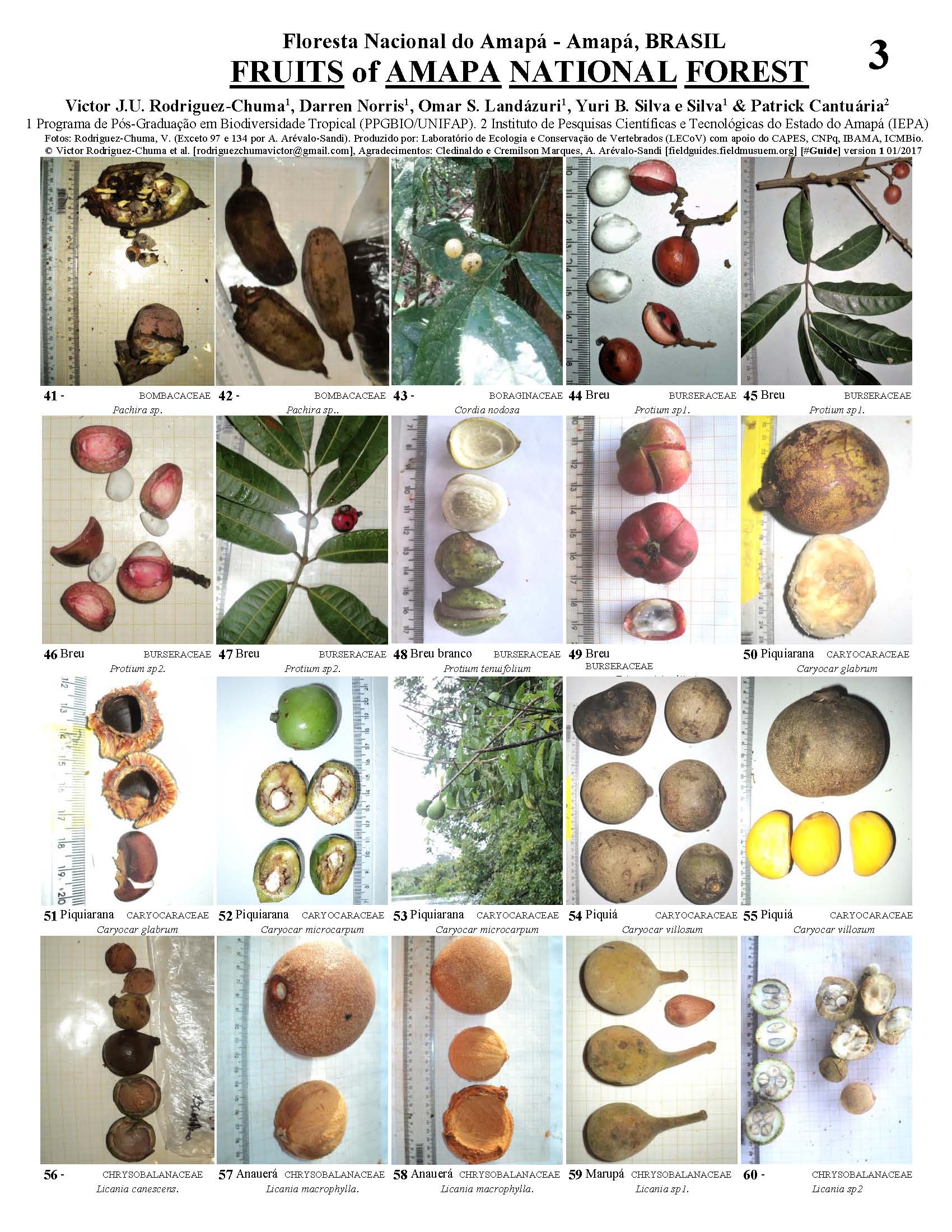


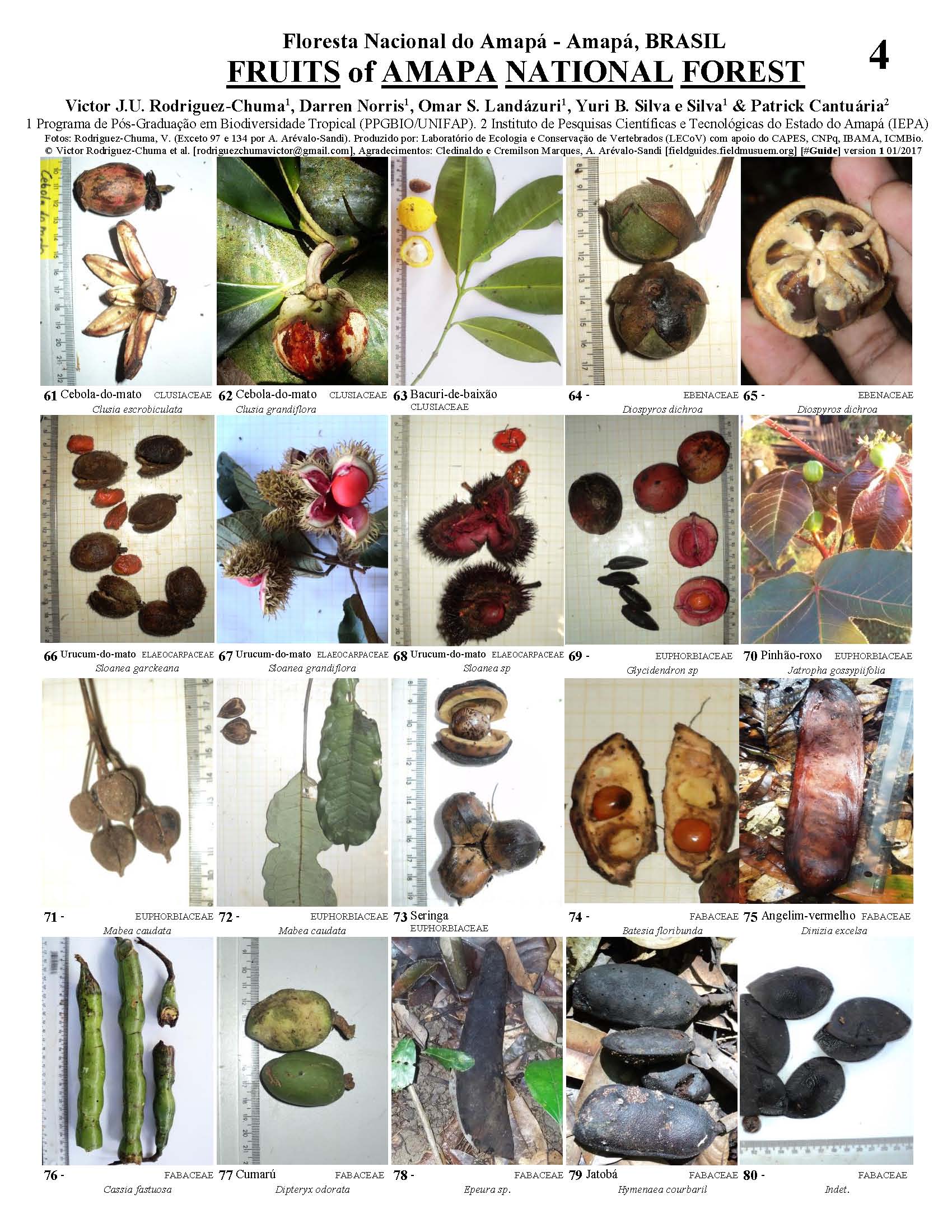


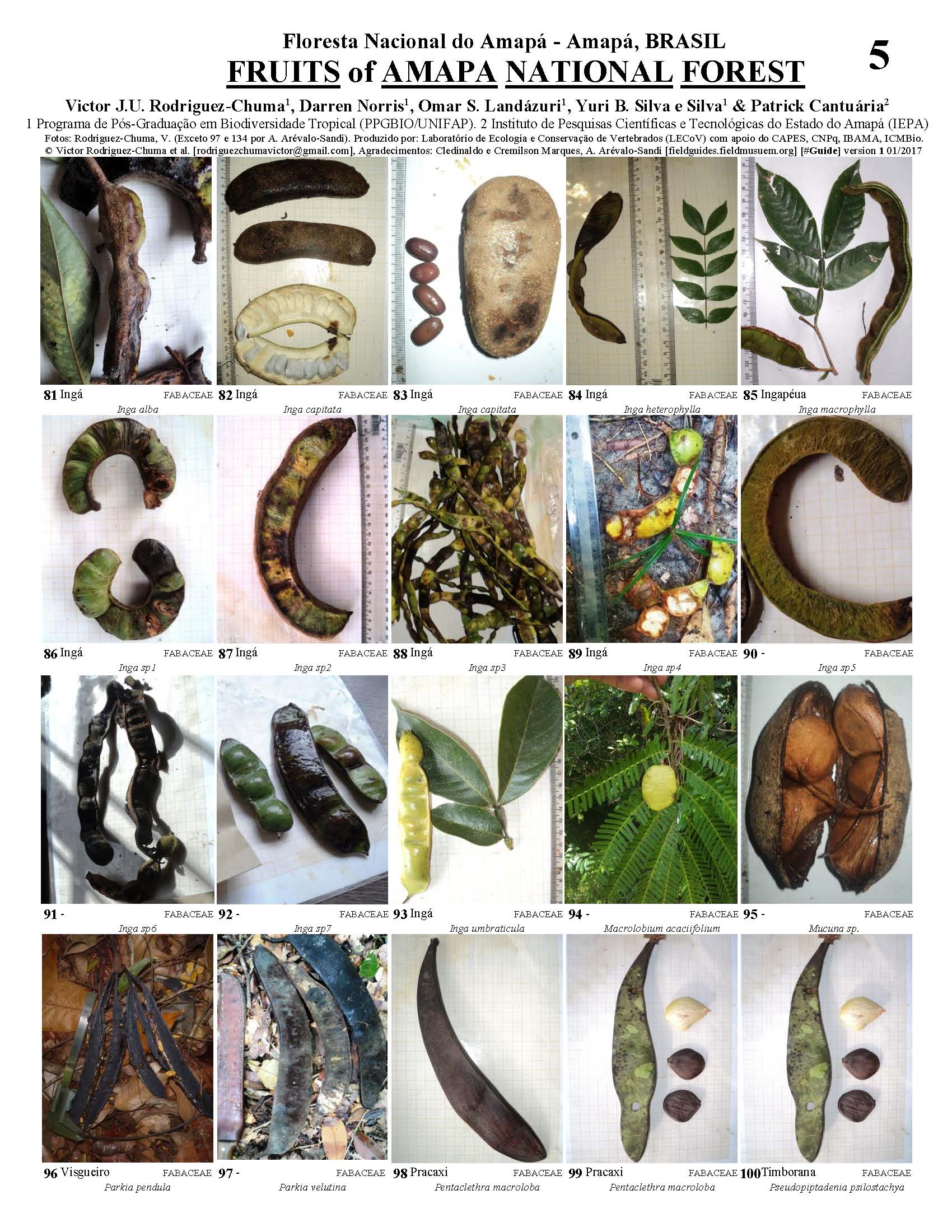


**Figure S4.** Mapped distribution (92-m spatial resolution) and histograms of explanatory variables used to explain fruit-fall patterns. See Table 1 main text for variable definitions.


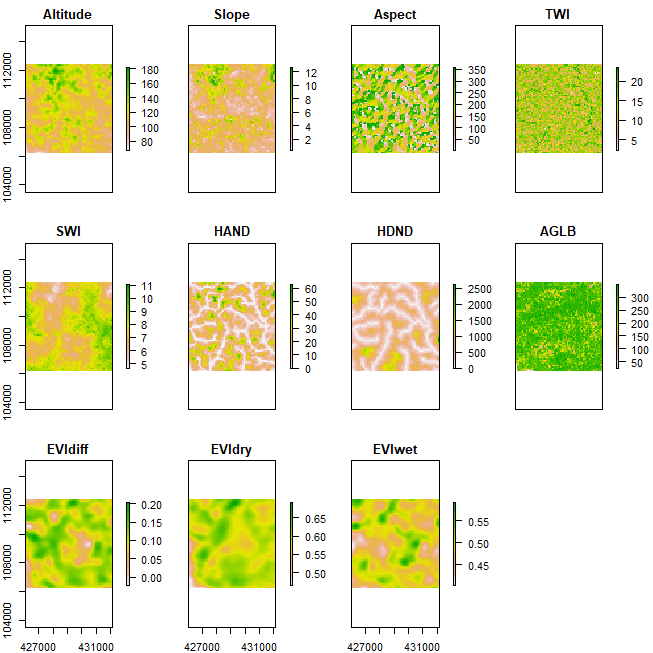

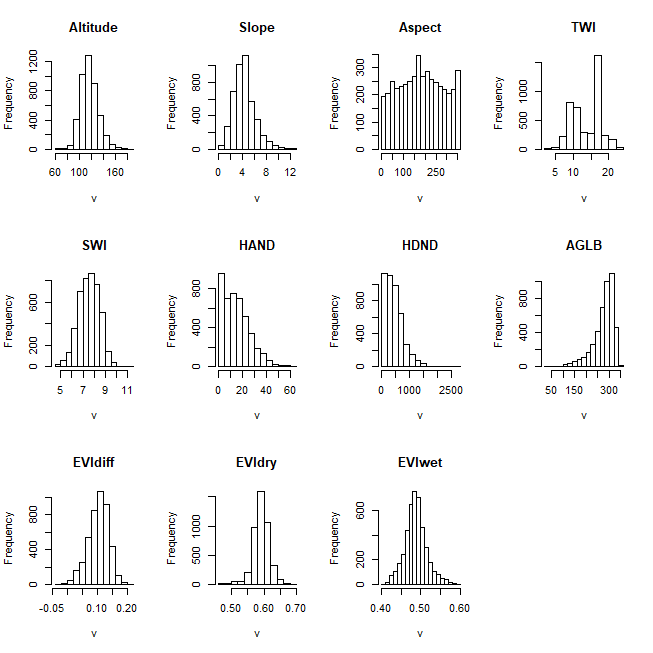

Supplement: Supplementary file 1 — Supplementary Information. [file 41598_2021_83803_MOESM1_ESM.docx]
